# A Method to Enhance Stroke Level of a MEMS Micromirror with Repulsive Electrostatic Force

**DOI:** 10.3390/mi11040401

**Published:** 2020-04-11

**Authors:** Niwit Aryal, Arezoo Emadi

**Affiliations:** Department of Electrical and Computer Engineering, University of Windsor, Windsor, ON N9B 3P4, Canada

**Keywords:** electrostatic actuation, fixed bottom electrodes, micro electromechanical systems, micromirror, repulsive electrostatic actuation, stroke

## Abstract

This paper presents a method to enhance the stroke level of a MEMS micromirror that, unlike conventional micromirrors, is actuated using a repulsive electrostatic force. The designed and proposed micromirror is held by L-shaped arms suspended over a set of bottom electrodes. In this configuration, three bottom electrodes are centered below each arm and are separated with a designed gap from each other to optimize the generated repulsive force. Using this approach, the micromirror surface is forced to deflect upward compared with the conventional downward deflection. The designed micromirror is proposed to utilize the PolyMUMPs fabrication technique from MEMSCAP Inc. In this work and in an unconventional approach, an air cavity of 2.75 µm can be achieved by combining the two available oxide layers through an additional removal of a polysilicon structural layer. It is shown that this design can significantly enhance the stroke level of the proposed micromirror to 5 µm at 150 V DC.

## 1. Introduction

Micro electromechanical systems (MEMS) micromirror is a candidate device to control the direction of a reflected light [1]. These devices can be actuated using several actuation mechanisms such as electrostatic [2,3], electromagnetic [4,5,6], and piezoelectric actuation [7,8,9,10]. Amongst the available actuation mechanisms, the electrostatic method is largely used due to several advantages including low power consumption, fast response time, and ease of fabrication [11,12]. Electrostatically actuated MEMS micromirrors are traditionally used in applications such as Light Detection and Ranging (LIDAR), optical coherence tomography, and micro-scanners [13,14]. However, a main disadvantages of the electrostatically actuated MEMS micromirror is their pull-in instability that limits the stroke the micromirror to 1/3 of the distance between the top and bottom actuating plates [15,16,17]. Stroke of these micromirrors is defined as the distance that the micromirror surface can travel in the space between the top and bottom actuating plates [12,16]. In a MEMS micromirror, a higher level of stroke can increase the device performance by improving the scanning angle [6,11]. Several methods are proposed to increase the stroke level of electrostatically actuated MEMS micromirror [18] including the introduction of comb-drive actuator structure that can lead to an improved out-of-plane stroke level of 1.2 µm for an input DC bias voltage of 60 V [2]. However, the comb-drive structure may add to the device fabrication complexity. In an attempt to increase the stroke of these micromirrors, their air cavity can be increased using custom fabrication processes [19,20], which may further increase the design complexity and cost of the device fabrication. In traditional parallel plate micromirrors, the electrostatically actuated devices developed using PolyMUMPs [21] fabrication technique can yield an out-of-plane stroke level of 1.65 µm at 100 V input DC bias voltage [22]. However, in this fabrication process the maximum air cavity that can be achieved is limited to 2 µm [21], which therefore, limits the downward deflection of the micromirror. 

In this work, an electrostatically actuated MEMS micromirror that benefits from repulsive electrostatic force is designed and simulated using Finite Element Analysis (FEA) method through COMSOL Multiphysics software. This proposed device employs a three-bottom electrode configuration [22,23] that generates an upward electrostatic force. This generated force is repulsive in nature, consequently, pushing the micromirror surface upward rather than conventional downward movement, and as a result reduces the pull-in instability effect of such systems. The proposed micromirror employs PolyMUMPs fabrication process where in an unconventional approach, the two oxide layers can be combined and as a result, an increased device air cavity of 2.75 µm can be achieved. This increased air cavity allows more space for the micromirror surface to travel, consequently increasing the stroke level. FEA simulations indicated that in this work, a high stroke level of 5 µm at 150 V DC is achieved for the designed device. 

## 2. Micromirror Design

### 2.1. Principal of Operation

**Figure 1:**
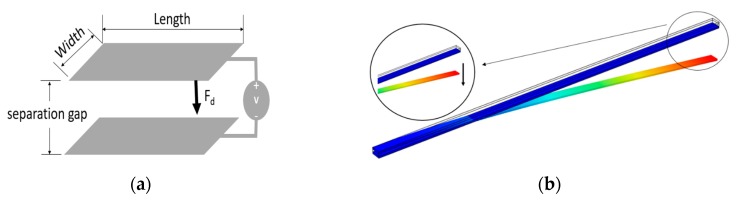
Schematic view of (**a**) a parallel plate electrostatic actuator and (**b**) an arm with one fixed bottom electrode showing the downward movement of the arm. The arm’s width, length, and voltage are 8 µm, 400 µm, and 50 V, respectively, whereas the width and length of the bottom electrode are 10 µm and 400 µm, respectively.

The proposed micromirror employs a parallel plate mirror structure held by L-shaped arms on all four sides of the micromirror [22,23] and is simulated using COMSOL Multiphysics. In a parallel plate micromirror model, when a DC voltage is applied between the two plates, the generated electrostatic force, *F_d_*, pulls the micromirror surface towards the bottom electrodes as shown in Figure 1a. Figure 1b illustrates a COMSOL simulation result of this downward deflection of the arm with one bottom electrode in a conventional approach. 

Ignoring the fringing field effects, this generated electrostatic force, *F_d_*, due to the applied voltage between the plates is given by Equation (1) [22]

(1) $$F_{d}=\frac{\varepsilon_{0}\;\varepsilon_{r}\;\;A\;\;V^{2}}{2d^{2}}\;,$$

where *ε*_0_ is the dielectric constant in air, *ε*_r_ is the relative permittivity of the medium, *A* is the overlapped area between the two plates, *V* is the applied voltage, and *d* is the gap between the two micromirror plates. This electrostatic force is a non-linear function of both voltage and the gap. When the micromirror plate is deflected to a distance of Δ*d* due to the applied voltage, *V*, Equation (1) can be re-written as [24]

(2) $$F_{d}=\frac{\varepsilon_{0}\;\varepsilon_{r}{\;A\;V}^{2}}{2{\left(d-\Delta d\;\right)}^{2}}.$$

Similarly, the micromirror plate and L-shaped arm spring force at the equilibrium are [22]

(3) $$F_{d}=-kd,$$

where *k* is the plate and arms’ spring constant. In conventional micromirrors and upon actuation, the top plate moves downward as shown in Figure 1. However, after reaching a displacement of *d* = −*d*_0_/3, this electrostatic force becomes larger than the micromirror plate’s restoring force, which drives the device to an unstable operating condition [16,25]. This phenomenon is known as pull-in instability that limits the stroke level of micromirror [25,26]. The pull-in voltage can be found from Equations (1) to (3) in (4):

(4) $$V_{pull-in}=\sqrt{\frac{8kd_{0}^{3}}{27\varepsilon_{0}\varepsilon_{r}A}}\;.$$

**Figure 2:**
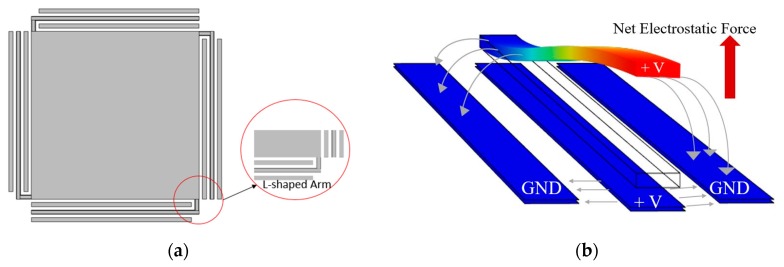
(**a**) Top view of the micromirror showing the L-shaped arm and three fixed bottom electrodes generated using COMSOL simulation software and (**b**) schematic view of the arm and the three bottom electrodes. COMSOL simulation results shows the distribution of generated electrostatic force and the upward movement of the. The device physical properties are provided in Table 1.

In order to reduce the pull-in instability effect, the proposed micromirror employs an L-shaped arm with three bottom electrodes as presented in Figure 2a. These three bottom electrodes are centered below each suspended arm to create a repulsive force when biased, which pushes the micromirror surface upward. These bottom electrodes are separated with a designed 8 µm distance from each other to create an optimum generated force as discussed in Section 2.2. The two side bottom electrodes are grounded, and the DC voltage is applied to the L shaped arm and the center bottom electrode. In this configuration, the net electrostatic force is pointed upward, consequently, pushing the micromirror surface upward rather than the conventional downward movement. The generation of the electrostatic force and the movement of the arm are illustrated in Figure 2b.

### 2.2. Micromirror Design Properties

The influencing physical properties of the designed micromirror are identified and their effects on the device stroke level are investigated and analyzed. COMSOL Multiphysics simulation software is utilized to tailor the device properties in order to achieve an enhanced stoke level. The simulations consider the limitations imposed by the employed PolyMUMPs fabrication technique [21] including layer thicknesses as well as minimum separation distance. These fabrication limitations are considered in the micromirror design. 

**Figure 3:**
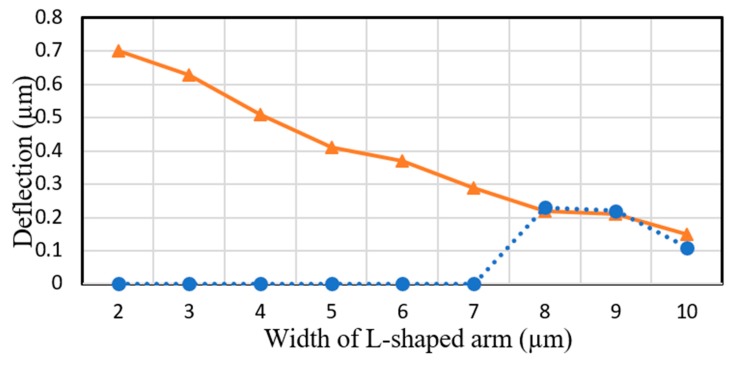
COMSOL simulation results illustrating deflection versus width of an individual arm without micromirror (solid line, triangle) and an L-shaped arm (dashed line, circle) connected to the micromirror.

In the proposed three bottom electrode configuration, it is shown that the width of the arm inversely affects the deflection amplitude as the effect of the fringing field that creates the upward deflection is reduced. COMSOL simulation results indicate a drop in an individual arm’s displacements when the width of the arm is increased from 2 µm, which is the minimum line width in PolyMUMPs process, to 10 µm as presented in Figure 3 (solid line, triangle). However, when the L-shaped arm is connected to a 400 µm by 400 µm micromirror, it is unable to lift the micromirror if the width of the arm is narrower than 8 µm as illustrated in Figure 3 (dashed line, circle). Therefore, in this proposed device, an L-shaped arm with the width of 8 µm is designed.

**Figure 4:**
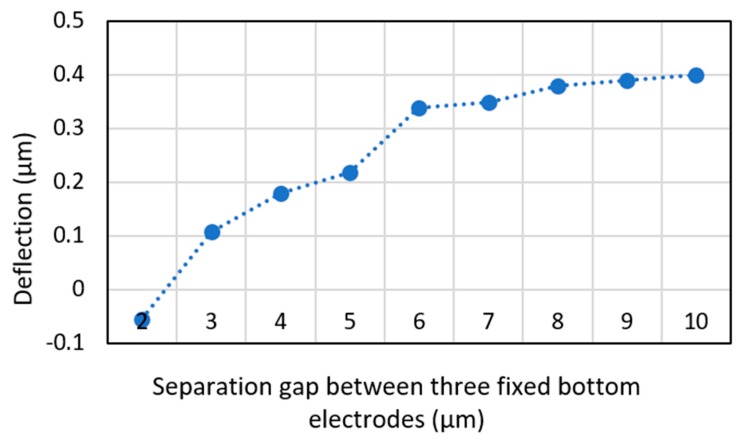
COMSOL simulation results illustrating deflection versus separation distance between fixed bottom electrodes for a micromirror with an L-shaped arm. The length and width of the L-shaped arm are 400 µm and 8 µm, respectively.

In addition to the width of the L-shaped arm, the separation distance between the bottom electrodes influences the deflection level of the arms and in return, the stroke level of the micromirror due to its effect on the device fringing field. COMSOL simulations are conducted when the separation distance between the bottom electrodes is increased from 1 µm to 10 µm, with a 1µm step size. The separation distance is not increased beyond 10 µm to minimize the overall area of the device. Figure 4 illustrates the simulation results and indicates that the stroke increases with an increase in the separation distance. Furthermore, the results show that when the separation distance is less than or equal to 2 µm, the upper arm moves downward that is undesired. Figure 4 shows that when the separation distance is increased beyond 8 µm, the increase in the deflection is insignificant. Therefore, for the proposed design the optimum separation distance between the fixed bottom electrode is selected equal to 8 µm. Table 1 presents the physical properties of the proposed micromirror.

**Figure 5:**
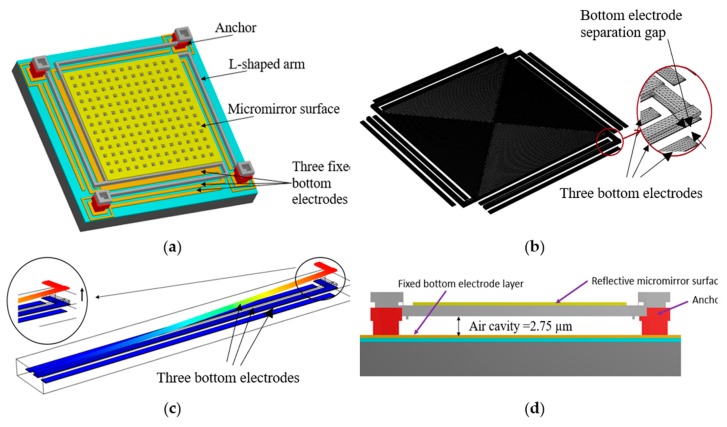
(**a**) Oblique view of the 400 µm by 400 µm micromirrors and (**b**) proposed micromirror simulated using COMSOL Multiphysics. (**c**) The schematic view of the L-shaped arm with three fixed bottom electrodes showing the upward movement of the arm and (**d**) cross-sectional view of the micromirror designed in MEMSPro using the PolyMUMPs fabrication technique.

In this work, the dimension of the reflective micromirror surface is 400 µm by 400 µm. The micromirror is connected to four L-shaped arms. The micromirror and the L-shaped arms are fabricated in the top doped polysilicon layer, P2, as shown in Figure 5a. The width and thickness of the L-shaped arms are 8 µm based on the simulations results obtained in Figure 3 and 1.5 µm based on the PolyMUMPs fabrication process, respectively. These four arms are connected to four anchors on all four sides of the micromirror as shown in Figure 5b. Each anchor that connects to the L-shaped arm and holds the micromirror is 50 µm in length and 50 µm in width. The three fixed bottom electrodes are made of the first doped polysilicon layer in PolyMUMPs, P0, with a thickness of 0.5 µm. These three fixed bottom electrodes are equal in dimension, which is 10 µm wide, 400 µm long and 0.5 µm thick, and are separated by an equal distance of 8 µm from each other based on the results obtained and presented in Figure 4. The corresponding upward deflection is shown in Figure 5c which consequently results in an upward movement of the micromirror attached to the L-shaped arm. A gap of 2.75 µm is achieved through combining the first and second oxide layers, O1 and O2, and by employing an additional etching steps to remove the middle polysilicon layer, P1, in the fabrication process. The cross-sectional view of the designed micromirror generated using MEMSPro is illustrated in Figure 5d. 

## 3. Device Fabrication

The designed micromirror is planned to be fabricated using PolyMUMPs fabrication process through MEMSCAP Inc. In this process, the polysilicon layers are used as the structural layers whereas the two available oxide layers are used as the sacrificial layers to create the cavity and separation between the top plate and the bottom electrode. This proposed device is designed based on utilization of seven main mask layers listed in Table 2 where the layer sequence and their thickness are predefined. 

**Figure 6:**
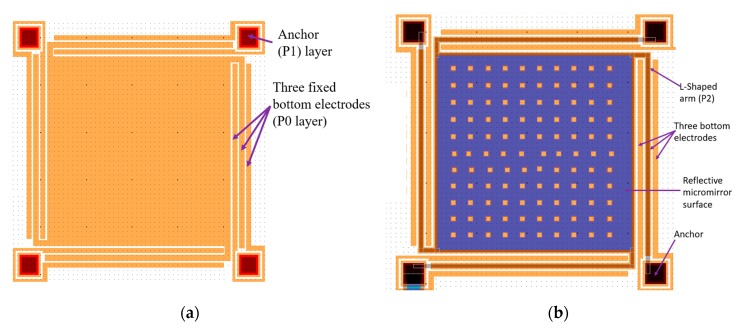
(**a**) Top view of the P0 and P1 designed layers and (**b**) the added P2 and Metal layers for the proposed micromirror in MEMSPro, using PolyMUMPs fabrication process.

The top views of the proposed micromirror with the additional etching layer that allows for a combined O1 and O2 and hence, larger cavity gap are presented in Figure 6a,b.

**Figure 7:**
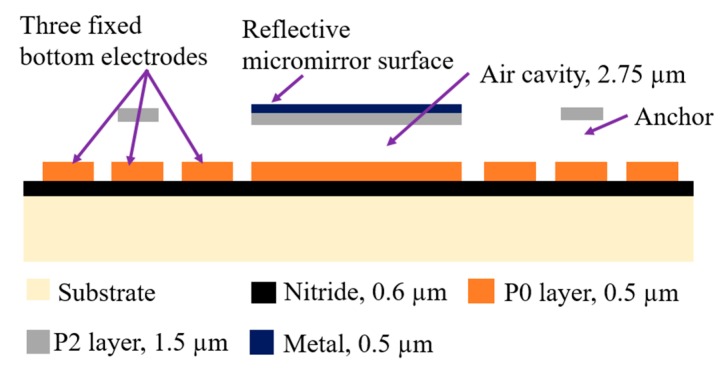
Cross-sectional view of the resulting micromirror.

The first layer in the PolyMUMPs fabrication process is an insulating layer of 0.6 µm low pressure chemical vapor deposition (LPCVD) silicon nitride that is deposited over a doped silicon substrate. The next layer is the first LPCVD polysilicon layer, P0, with a thickness of 0.5 µm that is patterned using POLY0 mask to create the fixed bottom electrode with the designed separation distance of 8 µm. The third layer is the first oxide layer, O1, which is 2 µm thick patterned and removed to open the anchor area using ANCHOR1 that is filled by P1 in the next step. This mask is negative and as such, this oxide layer remains on the rest of the device. The next structural layer is the second LPCVD polysilicon layer, P1, with a thickness of 2 µm patterned using Poly1. This mask layer is positive and therefore, the design shown in Figure 6a allows for the removal of P1 using a subsequent polysilicon etching. This layer is etched away in an attempt to combine the two available oxide layers to increase the gap. This step is then followed by the second oxide layer deposition, O2, with a thickness of 0.75 µm and patterning using POLY1_POLY2_VIA that opens an area on the anchor to be filled with P2 in the next step. The next layer is the third polysilicon layer, P2, which is 1.5 µm and is patterned to create the top plate as well as the L-shaped arms as shown in Figure 6b. The last deposited layer is the 0.5 µm gold layer that is deposited on the top plate and is used as the reflective surface of the micromirror. In this work, a gap of 2.75 µm is achieved through employing a combination of the two available sacrificial layers, O1 and O2 as described. The cross-section of the resulting device is illustrated in Figure 7.

For a fair comparison, two MEMS micromirrors with two different gap heights are designed and compared in this work. The second evaluated micromirror uses the conventional approach using PolyMUMPs process where only the first sacrificial layer, O1, is used to create a suspended top plate. Both the second deposited polysilicon, P1, and the second sacrificial layer, O2, are etched away on this design resulting in a gap height of 2 µm. All the other device physical properties, device configuration, as well as the structure of L-shaped arms and bottom electrodes remain the same. 

## 4. Design Evaluations

COMSOL Multiphysics is used to evaluate the designed micromirrors based on the POLYMUMPs fabrication process. Therefore, the designed MEMS micromirrors with the gap height of 2 µm and 2.75 µm are simulated and compared. 

**Figure 8:**
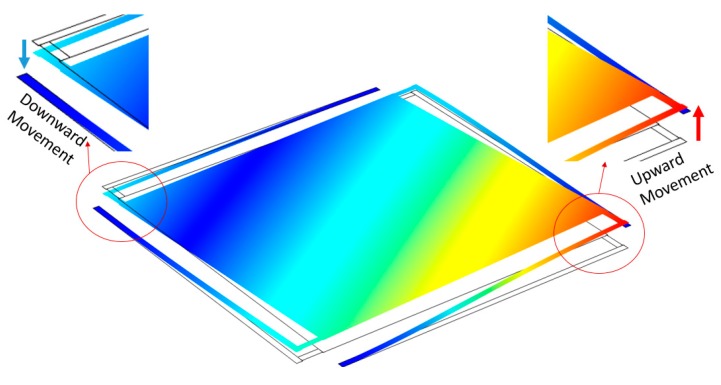
Schematic view of COMSOL simulation result showing the tilting of the micromirror surface where one end of the micromirror surface moves upward and the other end deflects downward with an unequal amplitude of deflection.

Simulations results shows that when a three bottom electrode configuration is utilized, a repulsive electrostatic force can be generated. The results indicate that one end of the micromirror can be pushed upward when a DC bias voltage is applied to the top membrane, one of the L-Shaped arms, as well as the center bottom electrode of that individual arm while the other bottom electrodes as well as the other three arms are grounded. Figure 8 illustrates the COMSOL simulation results of this configuration. As it can be seen in this figure, in this biasing condition the other end of the micromirror goes down which results in a larger overall displacement and angle. On the other hand, it is observed that in this configuration, the amplitude of the deflections on the two ends of the micromirror are not equal. Furthermore, in order to identify the maximum stroke and the uniformity of the surface, the amplitude of the deflection level along the diagonal cut-line on the micromirror surface is investigated. This investigation indicates that the micromirror surface tilts when it is actuated rather than buckling or bending as shown in Figure 8. 

Although the creation of the repulsive force and the upward movement of the biased end of the micromirror can eliminate the pull-in instability at that end, however, the forced downward deflection of the micromirror surface at the other end again limits the downward deflection to 0.91 µm. Therefore, it is important to note both upward and downward deflection and the new limitation of the device due to the pull-in instability on the downward moving side of the micromirror.

**Figure 9:**
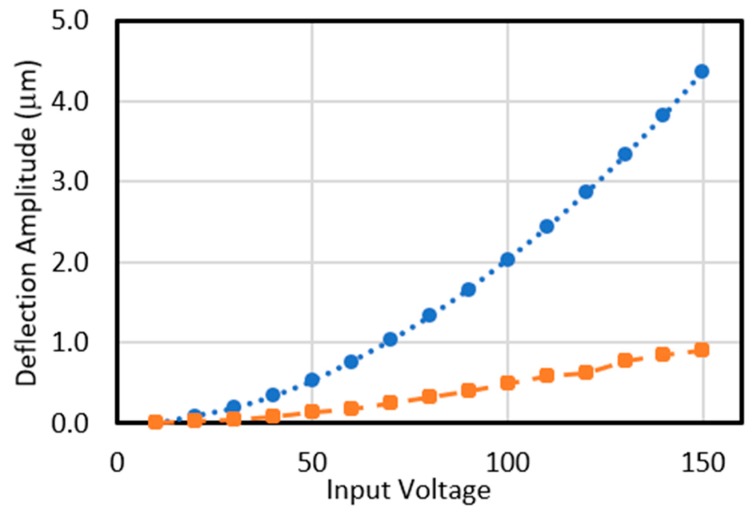
COMSOL simulation results presenting the effect of the input voltage on the upward (blue, circle) and downward (orange, square) deflections of the designed micromirror with a gap 2.75 µm.

The stroke of the designed MEMS micromirror with gap height of 2.75 µm is further simulated at various applied voltages and the upward (dashed line, circle) and downward (solid line, square) deflections’ amplitudes of the two sides of the micromirror are presented in Figure 9. The overall stroke of this micromirror is achieved by the difference of the upward and downward deflections. In this device, a maximum upward deflection of 4.3 µm is achieved at 150 V DC while the downward deflection of the other end of this micromirror is observed to be equal to 0.9 µm, below the pull-in instability limit. Therefore, a total stroke level of 5.2 µm is achieved for 150 V input DC bias voltage.

**Figure 10:**
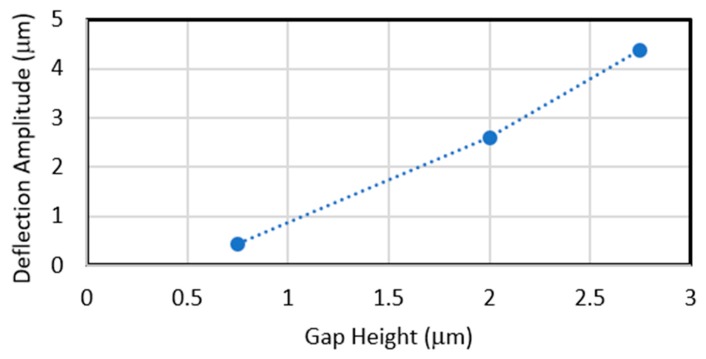
COMSOL simulation results presenting the effect of the gap height on the maximum upward deflections of the designed micromirrors.

A similar study is conducted for the conventional micromirror with a 2 µm gap height [22] and a micromirror employing only O2 as the sacrificial layer with 0.75 µm gap height. Their downward deflections are limited to 0.66 µm and 0.25 µm, respectively. Simulation results indicate that consequently, the micromirrors become unstable after 110 V and 60V DC, respectively. At these voltages, the maximum simulated micromirrors’ upward deflections are 2.6 µm and 0.43 µm, illustrated in Figure 10. A comparison between these results indicates that the stroke level is largely improved for the proposed micromirror with combined oxide layer with gap height of 2.75 µm compared with the conventional micromirror without the need for a complex fabrication process.

## 5. Conclusions

In this paper, a MEMS micromirror actuated using a repulsive electrostatic force with an enhanced stroke level is designed and simulated. Unlike the conventional micromirrors, the proposed micromirror employs a three bottom electrodes configuration, which pushes the micromirror surface upward rather than conventional downward movement. The micromirror is designed according to the PolyMUMPs fabrication process and by combining the two available oxide layers, the air cavity between the plates can be increased to 2.75 µm. A maximum stroke of more than 5 µm is achieved in this work for an input DC bias voltage of 150 V.

## Figures and Tables

**Table 1:** Physical properties of the micromirror with the air cavity of 2.75 µm.

Layer.	Length	Width	Height	Separation
Reflective Surface	400 µm	400 µm	0.5 µm	
Fixed Bottom Electrode	400 µm	10 µm	0.5 µm	8 µm
Arm	400 µm	8 µm	2 µm	
Anchor	50 µm	50 µm	2.5 µm	
Cavity			2.75 µm	

**Table 2:** Employed mask layers and their purposes in the PolyMUMPs fabrication technique.

Mask Layer	Purpose
POLY0	Pattern P0 layer and create the bottom electrodes, Figure 6a
ANCHOR1	Provide opening for P1 to be deposited on P0 on the four anchors of L-shaped arm by removing O1, Figure 6a
POLY1	Pattern P1 on only the four anchors of L-shaped arms, Figure 6a and allow for the etching of the P1 layer from the rest of the device area and deposition of O2 directly on O1
POLY1_POLY2_VIA	Provides opening for P1 to P2 connection by removing O2 from the four anchors of the L-shaped arms, Figure 6b
POLY2	Pattern P2 layer on four arms and the micromirror, Figure 6b
METAL	Pattern metal layer on the micromirror surface area, Figure 6b
HOLEM and HOLE2	Create releasing holes on metal and P2 layer to allow removal of O1 and O2
